# Role of Surgery in Potentially Resectable Small‐Cell Lung Cancer Based on the Eighth Edition of the TNM Classification: A Population Study of the US SEER Database

**DOI:** 10.1111/crj.70024

**Published:** 2024-10-21

**Authors:** Xiaokang Guo, Bin Wang, Jian Sun, Ji Li, Wenxiao Jia, Hui Zhu, Hongbo Guo

**Affiliations:** ^1^ Department of Thoracic Surgery, Shandong Cancer Hospital and Institute, Shandong First Medical University Shandong Academy of Medical Sciences Jinan Shandong China; ^2^ Department of Thoracic Surgery Changyi People's Hospital Weifang China; ^3^ Department of Radiation Oncology, Shandong Cancer Hospital and Institute, Shandong First Medical University Shandong Academy of Medical Sciences Jinan Shandong China

**Keywords:** lung cancer–specific survival, overall survival, SEER, small‐cell lung cancer, surgery

## Abstract

**Background:**

This study aimed to identify a specific SCLC population that would benefit from surgery.

**Methods:**

This study utilized patient data retrieved from the Surveillance, Epidemiology, and End Results (SEER) database spanning 2010 to 2017. To mitigate clinical biases, the propensity score matching (PSM) technique was employed. Separate cohorts were aligned using PSM according to the AJCC 8th edition TNM classification. The Kaplan–Meier method and a competing risk model were applied to evaluate overall survival (OS) and lung cancer–specific survival (LCSS), respectively.

**Outcomes:**

Among the 3394 patients with potentially resectable SCLC included in the study, 3062 underwent chemoradiotherapy and 332 underwent surgical treatment with adjuvant chemotherapy. Surgery was associated with better OS (median OS: 49 months; 95% CI: 35–63 months vs. 27 months; 95% CI: 21–33 months, *p* < 0.001) and LCSS (SHR, 0.578; 95% CI: 0.411–0.815, p < 0.001) in stage I patients after PSM. However, there was no significant difference in OS and LCSS between the surgery and nonsurgery groups in stage II and III patients after PSM. In the entire cohort, lobectomy was associated with improved OS (median OS: 48.6 vs. 28.7 months, *p* < 0.0001), but not LCSS (SHR, 0.696; 95% CI: 0.466–1.040, *p* = 0.078) compared with sublobar resection after PSM.

**Conclusion:**

Surgery with adjuvant chemotherapy significantly improved the survival prognosis of patients with early‐stage SCLC. However, surgical treatment should be carefully considered in patients with stage II/III disease. Lobectomy is oncologically equal to sublobar resection.

## Graphical abstract

This study supports a role for surgery in highly selected patients particularly in early SCLC. These results recommend surgery with adjuvant chemotherapy as first‐line therapy for early‐stage SCLC. However, surgical treatment should be carefully considered for stage II/III disease because of the similar outcomes to traditional treatment. We also found that lobectomy improved OS but not LCSS compared with sublobar resection in our study, which need more prospective to explore surgical methods for small‐cell lung cancer in the future.

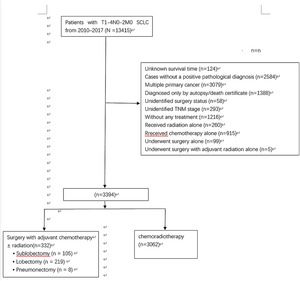

## 1 Introduction

Small‐cell lung cancer (SCLC) is a type of lung cancer characterized by rapid growth and high malignancy and is prone to mediastinal lymph node and distant metastasis unlike non–small‐cell lung cancer (NSCLC) [1]. SCLC constitutes about 15% of total lung cancer incidences [2]. Prior to 1970, SCLC was often treated using surgical resection. However, two randomized controlled trials on SCLC published in 1973 and 1994 reported no survival benefit of surgery [3, 4]. Chemotherapy in combination with radiation has become the standard treatment for SCLC. In recent years, an increasing number of retrospective studies based on large databases have supported the application of multimodal treatments, including surgery, for very early SCLC [5, 6, 7, 8, 9]. Currently, the National Comprehensive Cancer Network (NCCN) guidelines also recommend surgery for the comprehensive treatment of patients with T1‐2N0M0 SCLC [10]. We found that most of the research data originated prior to the 2010s. The potential impact of continuous and rapid advancements in surgical techniques, such as thoracoscopy and radiotherapy techniques, on survival outcomes remains unclear. Moreover, in current clinical practice, surgical treatment is often used only in the later stages, and whether surgery can benefit patients in later stages or achieve complete resection remain controversial. This study aimed to identify patients who may benefit from surgery and compare surgery types in a population with potentially resectable SCLC.

## 2 Material and Methods

### 2.1 Data Sources

This study retrospectively utilized data obtained from the Surveillance, Epidemiology, and End Results (SEER) database, which includes details on patients diagnosed with cancer from 2000 to 2018 (Incidence‐SEER Research Plus Data, 18 Registries, November 2020 Submission, 2000–2018). The SEER database is openly accessible and aims to contribute to the decrease of cancer incidences in the United States by providing statistical data on cancer. Information was gathered from 18 registries nationwide, representing about 28% of the population in the United States.

### 2.2 Study Population

The SEER program provided patient information including age at diagnosis, gender, ethnicity, tumor location, staging according to the American Joint Committee on Cancer (AJCC), and details on treatments like surgery, chemotherapy, and radiation. It also covered survival data, including the number of months survived, survival status, and specific causes of death according to SEER's classification. The study focused on patients diagnosed with stage T1‐4N0‐2M0 SCLC from 2010 to 2017, identified through the International Classification of Diseases for Oncology, third edition (ICD‐O‐3), using specific histology and topography codes. The codes 8041/3, 8042/3, 8043/3, and 8044/3 were employed to categorize tumors as small cell (not otherwise specified), oat cell, fusiform cell small cell, and intermediate cell small cell carcinomas, respectively. To ensure the dataset's integrity, patients with mixed histologies of NSCLC and SCLC, such as small cell‐large cell, small cell adenocarcinomas, small cell squamous cell carcinomas, and NSCLC with neuroendocrine features, were excluded.

Patients excluded from the surgical cohort included those with unclear surgical status, those who underwent surgery only, or those who received surgery followed by adjuvant radiation without chemotherapy. The nonsurgical group was strictly composed of patients treated with both radiation and chemotherapy. Additional exclusion criteria encompassed individuals with indeterminate survival duration, cases lacking a confirmed pathological diagnosis, subjects with more than one primary cancer, patients who did not receive any form of treatment, those with an unspecified TNM stage, and diagnoses made exclusively through autopsy or death certificates.

### 2.3 Statistical Analysis

Baseline demographics and clinical characteristics were analyzed using the Pearson *χ* ^2^ test for categorical variables. To assess overall survival (OS) and lung cancer–specific survival (LCSS), the study employed Kaplan–Meier survival curves along with log‐rank tests and competing risk analyses, respectively. The multivariate Cox regression model was applied to identify independent prognostic factors, calculating hazard ratios (HR) and 95% confidence intervals (95% CI). To adjust for potential confounders influencing lung cancer outcomes, propensity score matching (PSM) was utilized, generating scores through multiple logistic regression analysis considering variables such as age, gender, ethnicity, tumor location (laterality), primary site, overall TNM stage, T stage, and N stage. Matching was performed at a 1:2 ratio employing the caliper method with a tolerance of 0.02. This approach facilitated separate cohort comparisons to evaluate surgical benefits for stages I‐III patients. Statistical analyses were executed using SPSS (version 26.0; IBM Corp., Armonk, NY, USA) and Stata/SE (version 15.1; Stata Corp., College Station, Texas, USA). The significance threshold was set at a two‐tailed *p*‐value of <0.05. Subgroup analyses aimed at refining the study population and identifying subsets potentially deriving greater benefit from specific treatments. Kaplan–Meier and competing risk analyses for OS and LCSS were likewise applied to these subgroups.

## 3 Results

### 3.1 Patient Characteristics

**FIGURE 1:**
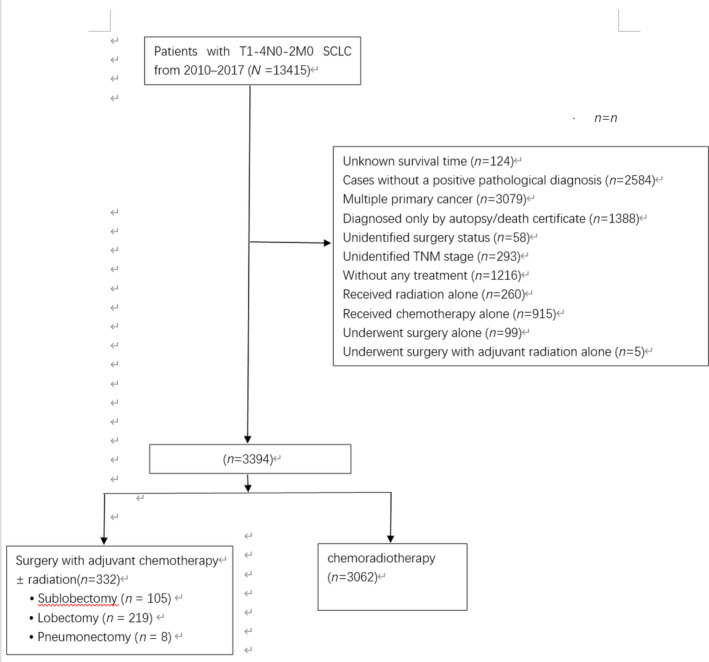
Study design (stage T1–4N2M0 patients).

Between 2010 and 2017, 13 415 patients were diagnosed with stage T1‐4N0‐2M0 SCLC (Figure 1). Of the 3394 patients that met inclusion criteria, 332 (9.78%) underwent surgery with adjuvant chemotherapy with or without radiation, and 3062 (90.22%) underwent radiotherapy and chemotherapy. In the surgery group, 219 patients underwent lobectomy, 105 underwent sublobar resection, and eight underwent pneumonectomy.

The baseline patient characteristics are presented in Table 1. More than half of the patients (54.60%) were aged between 60 and 64 years, and 1595 (55.83%) of the patients were female. Significant differences in race, primary site, TNM stage, T stage, and N stage were noted between the two groups. Compared with patients in the nonsurgical group, those in the surgical group had smaller tumor size (*p* < 0.001), mediastinal lymph node involvement (p < 0.001), and lower TNM stage (*p* < 0.001). PSM was performed for each cohort. The matched cohorts were well balanced in terms of basic demographics and clinical characteristics (Table 2).

**TABLE 1:** Baseline characteristics of patients before PSM.

Characteristic	All patients (*n* = 3394)	Surgical group (*n* = 332)	Nonsurgical group (*n* = 3062)	*p*
Gender				0.941
Male	1499	146	1353	
Female	1895	186	1709	
Age				0.454
<60	1001	88	913	
60–74	1853	189	1664	
75+	540	55	485	
Race				0.030
White	2889	297	2592	
Black	349	28	321	
Other	156	7	149	
Laterality				0.161
Left	1395	151	1244	
Right	1974	180	1794	
Unknown	25	1	24	
Primary site				<0.001
Upper lobe	1932	198	1734	
Middle lobe	169	15	154	
Lower lobe	747	108	639	
Overlapping lesion	42	3	39	
Main bronchus or carina or hilum or bronchus intermedius	303	3	300	
Not specified	201	5	196	
TNM stage				<0.001
IA	250	106	144	
IB	179	44	135	
IIA	292	59	233	
IIB	173	18	155	
IIIA	1837	98	1739	
IIIB	663	7	656	
T stage				<0.001
T1	767	171	596	
T2	1053	108	945	
T3	673	39	634	
T4	901	14	887	
N stage				<0.001
N0	775	178	597	
N1	435	66	369	
N2	2184	88	2096	

**TABLE 2:** Demographic characteristics of the matched cohorts.

	All patients			Stage I			Stage II			Stage III		
Characteristic	Surgery (*n* = 315)	NST (*n* = 546)	*p*	Surgery (*n* = 121)	NST (*n* = 174)	*p*	Surgery (*n* = 73)	NST (*n* = 130)		Surgery (*n* = 87)	NST (*n* = 170)	*p*
Gender			0.387			0.973			0.597			0.887
Male	137	221		54	78		32	62		34	68	
Female	178	325		67	96		41	68		53	102	
Age			0.897			0.871			0.519			0.609
<60	76	138	p	20	25		17	37		21	51	
60–74	187	323		78	114		47	73		50	91	
75+	85	85		23	35		9	20		16	28	
Race			0.631			1			0.608			1
White	281	475		121	174		64	117		87	177	
Black	27	57		—	—		9	13		—	—	
Other	7	14		—	—		—	—		—	—	
Laterality			0.916			0.895			0.889			0.805
Left	143	254		54	79		38	69		38	77	
Right	171	289		67	95		35	61		49	93	
Unknown	1	3		—	—		—	—		—	—	
Primary site			0.401			0.804			0.896			0.642
Upper lobe	187	346		76	117		40	68		110	56	
Middle lobe	14	21		5	8		4	10		—	1	
Lower lobe	103	153		39	47		28	49		52	26	
Overlapping lesion	3	2		—	—		—	—		—	—	
Main bronchus	3	9		1	2		—	—		2	2	
Not specified	5	15		—	—		1	3		6	2	
TNM stage			0.358			—			—			—
IA	89	123		—	—		—	—		—	—	
IB	44	71		—	—		—	—		—	—	
IIA	59	101		—	—		—	—		—	—	
IIB	18	44		—	—		—	—		—	—	
IIIA	98	194		—	—		—	—		—	—	
IIIB	7	13		—	—		—	—		—	—	
T stage			0.531			0.248			0.498			0.763
T1	154	242		85	111		30	43		64	31	
T2	108	199		36	63		27	57		51	28	
T3	39	72		—	—		16	30		27	17	
T4	14	33		—	—		—	—		28	11	
N stage			0.384			1			0.892			0.949
N0	161	271		121	174		22	38		5	11	
N1	66	100		—	—		51	92		7	15	
N2	88	175		—	—		—	—		75	144	

### 3.2 OS Analysis

**FIGURE 2:**
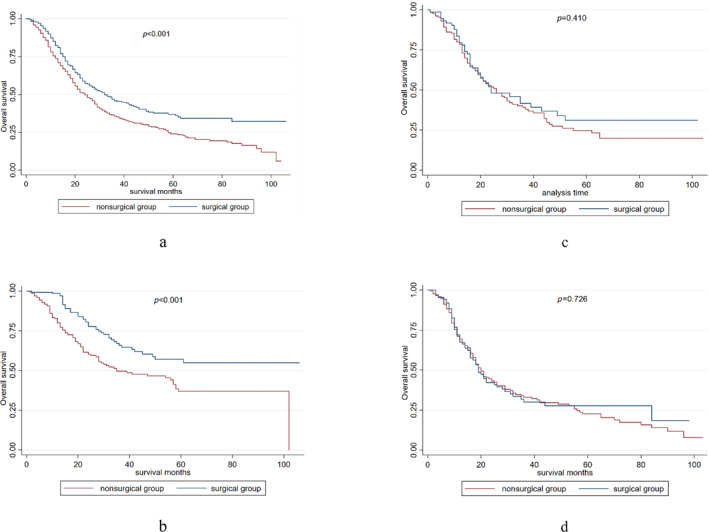
(a) The OS curve of the whole cohort after PSM. (b) The OS curve of the stage I SCLC after PSM. (c) The OS curve of the stage II SCLC after PSM. (d) The OS curve of the stage III SCLC after PSM.

Patients treated with surgery had significantly longer OS compared with those without surgery in the entire cohort (median OS: 32 months; 95% CI: 25–39 months vs. 22 months, 95% CI 19–25 months, *p* < 0.001) (Figure 2a). Figure 2b–d shows the OS results after PSM according to the different TNM stages. Surgery was associated with longer survival only in patients with stage I SCLC (median OS, 49 months; 95% CI, 35–63 months vs. 27 months, 95% CI, 21–33 months, *p* < 0.001). Surgery did not provide any survival benefit in patients with stage II (median OS, 24 months; 95% CI: 19–43 months vs. 26 months, 95% CI: 20–32 months, *p* = 0.410) or stage III (median OS, 19 months; 95% CI: 16–26 months vs. 20 months, 95% CI: 17–25 months, *p* = 0.728) SCLC.

### 3.3 LCSS Analysis

**FIGURE 3:**
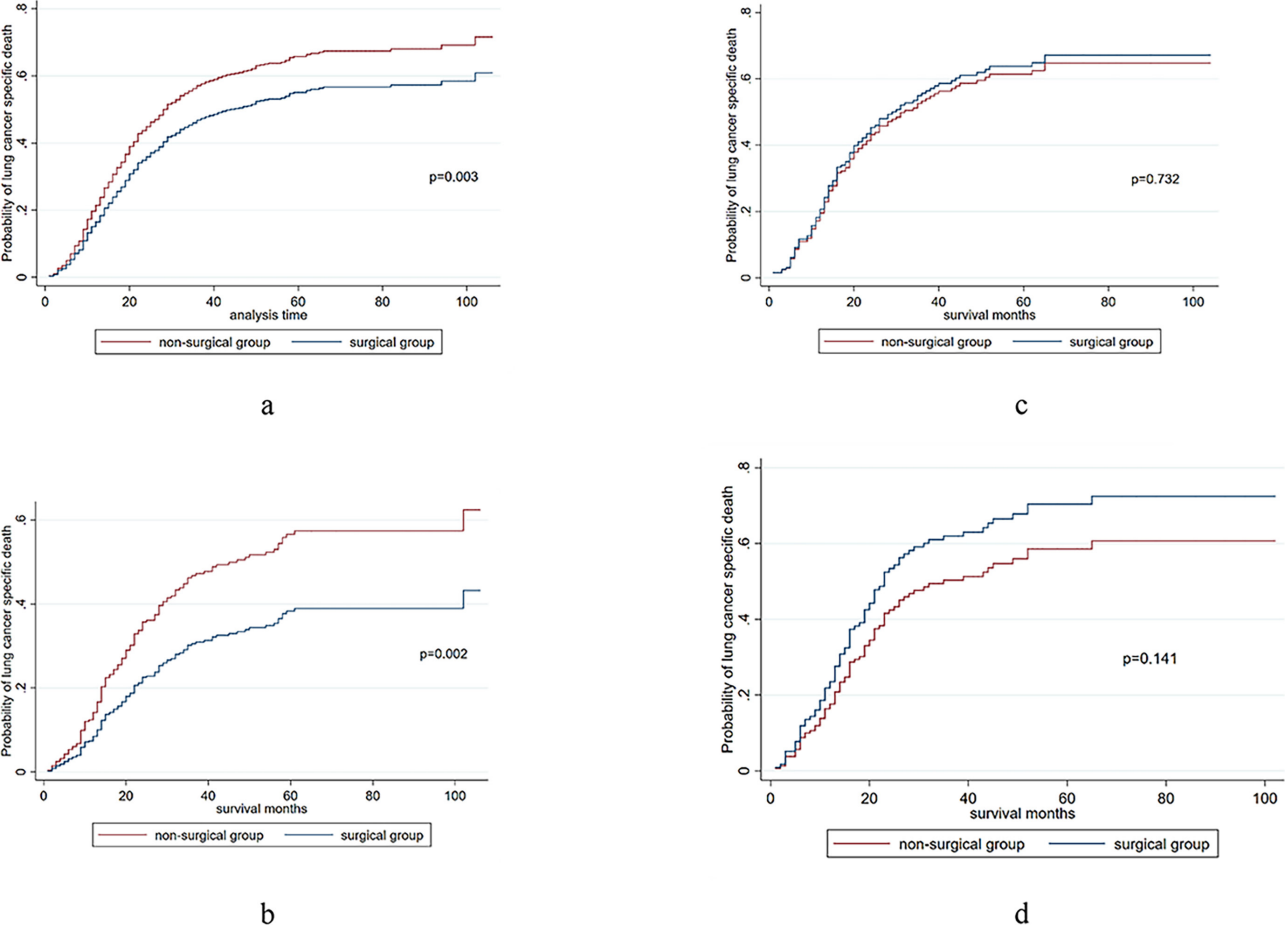
(a) The LCSS curve of the whole cohort after PSM. (b) The LCSS curve of the stage I after PSM. (c) The LCSS curve of the stage II SCLC after PSM. (d) The LCSS curve of the stage III SCLC after PSM.

A competing risk analysis–based cumulative incidence curve for LCSS after PSM for all patients is shown in Figure 3a. The surgical group showed a higher probability of LCSS compared with the nonsurgical group after PSM (SHR: 0.919; 95% CI: 0.845–0.999, *p* < 0.001). The competing risk analysis–based cumulative incidence curves for LCSS were also generated for the subgroups (Figure 3b–d). Probability of LCSS was higher in the surgical group than that in the nonsurgical group for stage I patients (SHR: 0.578; 95% CI: 0.411–0.815, *p* < 0.001). There was no significant difference in LCSS between the two groups in stages II (SHR: 1.068, 95% CI: 0.734–1.552, *p* = 0.984) and III (SHR: 1.017, 95% CI: 0.729–1.420, *p* = 0.974) patients.

### 3.4 Survival Analysis Based on the Type of Surgery

**FIGURE 4:**
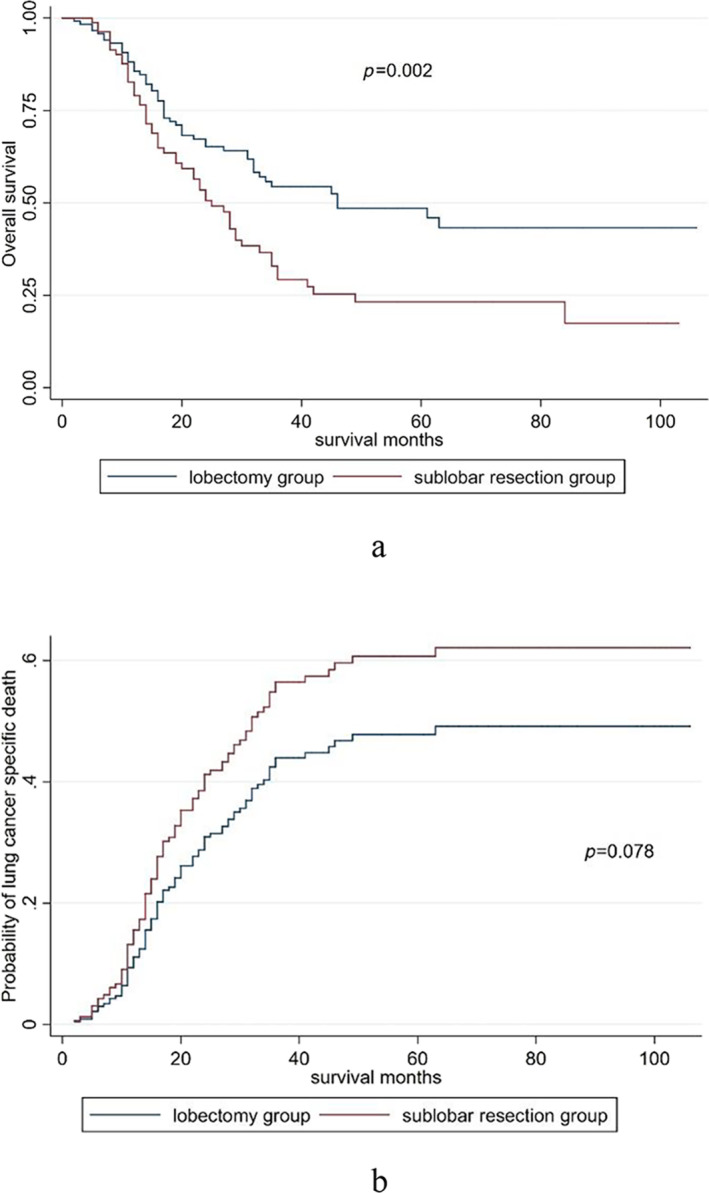
(a) The OS curve between lobectomy and sublobar resection groups after PSM. (b) The LCSS curve between lobectomy and sublobar resection groups after PSM.

Eight pneumonectomy cases were excluded from the comparison. The baseline characteristics of the lobectomy and sublobar resection groups after PSM are shown in Table 3. The results showed that lobectomy significantly improved the OS compared with sublobar resection (median OS: 46 months, 95% CI, 21–71 months vs. 25 months, 95% CI: 19–30 months, *p* = 0.002) (Figure 4a). Lobectomy showed a trend toward higher LCSS compared with sublobar resection, but the difference was not significant (SHR, 0.696; 95% CI: 0.466–1.040, *p* = 0.078) (Figure 4b).

**TABLE 3:** Baseline characteristics of the lobectomy and sublobar resection after PSM.

Characteristic	All patients (*n* = 199)	Lobectomy (*n* = 118)	Sublobectomy (*n* = 81)	*p*
Gender				0.822
Male	89	52	37	
Female	110	66	44	
Age				0.759
<60	51	29	22	
60–74	114	67	47	
75+	34	22	12	
Race				0.876
White	118	110	74	
Black	11	6	5	
Other	4	2	2	
Laterality				0.429
Left	94	53	41	
Right	105	65	40	
Unknown	—	—	—	
Primary site				0.332
Upper lobe	59	85	59	
Middle lobe	3	1	3	
Lower lobe	19	32	19	
Overlapping lesion	—	—	—	
Main bronchus	—	—	—	
Not specified	—	—	—	
TNM stage				0.712
IA	79	43	36	
IB	31	18	13	
IIA	23	15	8	
IIB	8	6	2	
IIIA	58	36	22	
IIIB	—	—	—	
T stage				0.499
T1	118	68	50	
T2	63	37	26	
T3	18	13	5	
T4	—	—	—	
N stage				0.632
N0	122	70	52	
N1	22	15	7	
N2	55	33	22	

## 4 Discussion

Since the 1970s, radiotherapy and chemotherapy have replaced surgery as the standard treatments for limited‐stage SCLC (L‐SCLC). Surgery was previously rejected as a treatment option based on the results of two randomized controlled trials. In 1973, the Medical Research Council (MRC) published the 10‐year follow‐up results of a randomized controlled study comparing surgery and radiotherapy in SCLC, in which surgery did not improve survival compared with radiation [3]. In 1994, the Lung Cancer Group carried out a prospective randomized trial on whether surgery should be performed on residual lesions following chemoradiotherapy in SCLC and showed that surgery was not associated with better survival (median survival times of 15.4 and 18.6 months, respectively, and 2‐year OS rate of approximately 20% [*p* = 0.78] in both groups) [4]. Neither study supported surgical option in SCLC. However, these two studies had some limitations. Only 48% of patients underwent “complete resection” in the surgery group, and 34% underwent exploratory or palliative surgery, which reduced the value of surgery in the study published by MRC. Although all the patients enrolled in Lung Cancer Group had LD‐SCLC, only 49% (29/70) of the patients did not have lymph node metastasis, and there was no stratified study on the prognosis of early‐stage SCLC. In addition, these two studies were performed a few decades ago, when diagnostic imaging methods were not as advanced, and modern techniques such as positron emission computed tomography (PET‐CT), CT, and magnetic resonance imaging (MRI) did not exist. Therefore, the staging was likely inaccurate. In 1994, Liao et al. conducted a randomized controlled study that included 40 patients with stage III SCLC who were randomly divided into chemotherapy (CT)‐surgery‐CT and CT‐radiotherapy (RT)‐CT. The 2‐year survival rate of the CT‐surgery‐CT group was significantly better than that of the CT‐RT‐CT group, supporting the surgical approach [11]. In 2018, Liu's systematic analysis, which compiled data from three studies involving 330 individuals, revealed that surgery did not offer a notable advantage over traditional treatments [12]. However, there was high heterogeneity among the three studies, and the quality of evidence was judged to be “very low” for all outcomes.

Some studies have shown the local recurrence rate of SCLC to be as high as 20%–65% following radiotherapy and chemotherapy. Autopsies showed that 92% of the SCLC tumor remained in nonsurgical patients, whereas only 31% of the tumor remained in radical surgery patients. Clearly, surgery plays an irreplaceable role in eradicating primary lesions, reducing local recurrence, and ultimately improving long‐term survival. An increasing number of retrospective studies based on large databases, such as SEER and NCBD, have shown that surgery offers advantages in early SCLC [5, 6, 7, 8, 9]. In 2010, Varlotto et al. showed that lobectomy alone was superior to sublobar resection and radiotherapy alone for stage I patients based on the SEER database [5]. In 2017, Yang et al. stratified patients according to surgery, chemotherapy, and brain‐preventive radiotherapy to assess the outcomes based on the NCBD database. They recommended that patients with pT1‐2N0M0 stage SCLC should be treated surgically, and reported the order of curative effects of postoperative treatment to be as follows: surgery + postoperative adjuvant chemotherapy + brain‐preventive radiotherapy, surgery + postoperative adjuvant chemotherapy, surgery + chest radiotherapy, and nonsurgical treatment [6]. However, current evidence is insufficient to consider surgery in patients with locally advanced disease in later stages. Casiraghi et al. advice that the surgical option should be considered carefully in patients with stage II and III disease, given the poor survival outcomes in such patients [13]. However, a few recent studies have reported that surgery may provide some survival benefits for locally advanced patients; however, the survival benefits are significantly lower compared with that in patients with stage I SCLC [14, 15, 16].

To identify the subpopulation that may benefit from surgery, we performed a subgroup analysis based on TNM stage. Our results showed that comprehensive treatment, including surgery, can improve OS and LCSS only in patients with stage I patients, which is consistent with the recommendations of the NCCN guidelines. Surgery did not confer a survival advantage compared with chemoradiotherapy in patients with advanced stage, which is contrary to some studies [14, 15, 16]. Casiraghi et al. showed that lymph node metastasis and T stage significantly affect OS. Patients diagnosed with pT4 or pN2 classifications faced the most severe prognosis, exhibiting 1‐year survival rates of 50% and 57.1%, respectively, and 5‐year survival rate of 0% [13]. These data suggest that SCLC remains a highly malignant, easily metastatic systemic disease in which local benefit from surgery may not translate into a survival advantage for patients with locally metastatic disease.

Consistent with the results of some previous studies, our data suggest that lobectomy improves OS compared with sublobar resection [5, 14, 15, 16, 17]. However, contrary to previous data from the SEER database showing that lobectomy is superior to sublobar resection in early or locally advanced cases, lobectomy did not improve LCSS in our study [5, 17]. These studies included competing events as censored data to calculate LCSS, which may have caused some bias. In clinical practice, sublobar resection is often performed in patients with poor lung function and physical status, and survival is often affected by other diseases. Our data also suggest that the probability of non–lung cancer–related death was higher in patients who underwent sublobar resection than in those who underwent lobectomy. This may explain why lobectomy improved only OS and not LCSS when we used a competing risk model to exclude the impact of non–tumor‐related death. In addition, the circumstances under which pneumonectomy is recommended for SCLC are uncommon, and it is generally considered as a salvage surgery [18, 19, 20]; only eight patients underwent pneumonectomy in our study, which is significantly lower than the number of patients who underwent pneumonectomy in the previous SEER database. Given its high risk of postoperative complications and poor prognosis, this approach is not currently recommended.

Although this was a retrospective study, we included a large number of patients, which ensured sufficient power to draw conclusions regarding relative survival benefits. We used PSM to control for bias due to the large heterogeneity between the groups. We used a competing risk model to calculate the LCSS, making the results statistically more accurate.

This study had some limitations. Firstly, although we applied the PSM method to reduce selection bias as much as possible, we could only balance the available variables and not potentially unknown factors that may have affected the results. Secondly, the SEER database lacks comprehensive details on chemotherapy or radiotherapy protocols, patient performance status, complications, and disease‐free survival. Thirdly, the patient count in the surgical cohort was notably less than that in the nonsurgical group, potentially reducing the statistical significance of the analysis.

## 5 Conclusion

This research endorses the application of surgical intervention in certain individuals, especially those diagnosed with early‐stage SCLC. The findings are in line with existing guidelines that advocate for surgery accompanied by adjuvant chemotherapy as the primary treatment for stage I SCLC. Furthermore, our results reinforce the need for randomized studies on multimodal therapies, including surgery, for the treatment of early SCLC. Lobectomy improved OS, but not LCSS, compared with sublobar resection. Further studies are needed explore surgical methods for SCLC in the future.

## Author Contributions

Xiaokang Guo was responsible for data collection, statistical analysis, and the drafting and revision of the manuscript. Bin Wang and Jian Sun focused on data gathering, summarization, and statistical evaluation. Ji Li and Wenxiao Jia were tasked with literature search and provided assistance in both writing and editing the manuscript. Hongbo Guo and Hui Zhu played a crucial role in reviewing, editing the manuscript, and offering guidance on the article's composition. All authors made substantial contributions to the work and gave their approval for the final version to be published.

## Ethics Statement

This research has been approved by the ethics committee of Shandong Cancer Hospital and Institute. This study utilized deidentified data stored in the SEER database and, therefore, waived the requirement for individual informed consent.

## Conflicts of Interest

The authors declare no conflicts of interest.

## Data Availability

Publicly available datasets were analyzed in this study. These data can be found here: www.seer.cancer.gov.
